# Older Adults’ Socio-Demographic Determinants of Health Related to Promoting Health and Getting Preventive Health Care in Southern United States: A Secondary Analysis of a Survey Project Dataset

**DOI:** 10.3390/nursrep11010012

**Published:** 2021-02-15

**Authors:** Huey-Ming Tzeng, Udoka Okpalauwaekwe, Chih-Ying Li

**Affiliations:** 1 School of Nursing, The University of Texas Medical Branch, Galveston, TX 77555, USA; 2 College of Medicine, University of Saskatchewan, Saskatoon, SK S7N 2Z4, Canada; udokaokpala.uo@usask.ca; 3 Department of Occupational Therapy, School of Health Professions, The University of Texas Medical Branch, Galveston, TX 77555, USA; chili@utmb.edu

**Keywords:** older adults, health promotion, disease prevention, self-care

## Abstract

**Background:** This exploratory survey study examined the relationship between older adults’ five socio-demographic determinants (urban/rural residence, gender, age, marital status, and education) and their self-reported perception of importance, desire to perform, and ability to perform nine self-care behaviors related to promoting health and getting preventive health care. **Methods:** We reported a secondary analysis of a dataset from an exploratory survey project; we analyzed 2015–2016 retrospective data collected from a cross-sectional survey study, including 123 adults aged 65 years and older living in southern United States. Data were collected from the Patient Action Inventory for Self-Care and a demographic questionnaire and analyzed using binary and multiple logistic regression analyses. **Results:** Advancing age, marital separation, and holding less than a high school education were significantly associated with at least one of the unfavorable perceptions of the importance, the desire to perform, and the ability to perform three self-care behaviors. These three behaviors were: (1) creating habits that will improve health and prevent disease, (2) discussing the use of health screening tests with healthcare providers, and (3) joining in local health screening or wellness events. Gender and urban/rural residence were not significant. **Conclusions:** Comprehensive health care should include an individual’s socio-demographic context and self-care perception of importance, desire, and ability.

## 1. Introduction

Social determinants of health are complex and inter-related, which require multilevel approaches to eliminate health disparities such as environmental and occupational lunch disease and accessing diagnostic testing [1,2]. Accessing health promotion and preventive health care (e.g., laboratory and public health services) during the COVID−19 pandemic is ever more challenging [2]. Social determinants of health, insurance status, health care providers’ bias, and other social influences have been shown to impede patient-centered care delivery [3]. Health care and social service providers’ understanding of socio-demographic determinants of health can improve overall health metrics in the United States [4].

The development of effective patient education requires individualized tailoring. For example, a home-based computerized cognitive training program with adaptive difficulty and individual tailoring showed better memory and learning improvement than more generic cognitive training [5]. Lacsamana et al. [6] suggested that governments, health systems, and non-government funding agencies seek community-based solutions to resolve health problems’ root causes by targeting socio-demographic determinants. Lacsamana et al. further recommended strengthening communities’ resilience to pursue fundamental health system changes for better health promotion and disease prevention [6]. A recent U.S. study [7] found that community-dwelling adults (of which 53.7% were aged 65 years and older) who reported being able to perform health management behavior (related to following up on health screening results) were less likely to have emergency room visits. Following up on health screening results is a behavior related to getting preventive services defined by the Center for Advancing Health and Tzeng and Pierson’s study [8,9]. This finding underlined the significance of older adults empowering themselves to perform actions associated with health promotion and disease prevention [7,8,9].

### 1.1. Purpose of the Study

As a result, this exploratory survey study investigated the relationships between five socio-demographic determinants and the perception of importance, desire to perform, and ability to perform nine self-care behaviors related to promoting health and getting preventive services among community-dwelling older adults living in the southern United States. The five demographic traits are urban/rural residence, gender, age, marital status, and education. The first four self-care behaviors are related to promoting health, and the other five behaviors are associated with getting preventive services [7,8,9] (see Section 2.2). We conducted a secondary analysis of a dataset from an exploratory survey project.

The overarching research question was: What are the socio-demographic determinants of older adults’ perceptions and ability to perform self-care behaviors related to promoting health and getting preventive health care? Our findings can be used to develop community-based solutions to address the distinctive needs of older adults. The rationale was that medical and clinical strategies alone could not produce healthier outcomes without addressing the root causes of health problems and targeting socio-demographic determinants of health behaviors [6].

### 1.2. Background

#### 1.2.1. Importance of the Study

As indicated in the report of the *2018 Profile of Older Americans* [10], the population group aged 65 years and older increased by 34% between 2007 and 2017. The aging populations are projected to grow 86% between 2017 and 2060 in the United States. In 2017, 15.6% of the U.S. population was aged 65 years and older. The life expectancy for this group is 85.6 years old for females and 83.1 years for males. A more recent data showed that in the first two quarters of 2018, 20% of older Americans aged 85 years and older needed help with personal care, compared to 9% for adults aged 75–84 years old and 4% for adults aged 65–74 years old [10]. Older adults have self-care needs related to health promotion, disease prevention, and assistance in maintaining independence increase with advancing age, especially for women and women who live alone [10,11,12].

To the authors’ best understanding, limited studies have explored the association between community-dwelling older adults’ self-care needs related to health promotion and disease prevention and their demographic characteristics [7,8,9,13]. No similar studies have been reported in the United States and other countries. A brief literature review was conducted and summarized in Section 1.2.2.

#### 1.2.2. Summary of the Literature Review

We conducted a literature search on PubMed between 20 September 2019–30 September 2019. We used the following keyword syntax: “older adult” AND “health promotion” OR “promoting healthy habits” OR “promoting healthy behavior” OR “living healthy” OR “support service utilization” OR “patient compliance” OR “patient adherence” OR “medical adherence” OR “preventive care” OR “early cancer screening” OR “social participation” AND “demographic characteristics” OR “determinants of health” OR “determinants of health.” The search strategy was limited to journal articles, reviews, and systematic reviews published within a five-year timeframe (1 January 2014, to 30 September 2019). This search resulted in an initial pool of over 7000 articles. Based on our study interest through multiple online meetings involving the first two authors, further refining resulted in 23 articles that addressed the demographic characteristics of interest. The article selection strategy included the clarity of research objectives, i.e., exploring the relationships of five socio-demographic determinants (urban/rural residence, gender, age group, marital status, and education) with the healthy lifestyle and disease prevention self-care behaviors examined in this study. The 23 identified articles that addressed or implied a linkage between any of the five socio-demographic determinants and the self-care behaviors related to promoting health or getting preventive health care. The findings were as follows:

*Residence*: Two studies [14,15] found that urban dwellers were more likely to adopt healthy habits and receive preventive health care than their rural counterparts. However, we found two articles with the contrast finding [16,17].

*Gender*: Eight studies [14,16,18,19,20,21,22,23] reported that female older adults were more likely to perform health promotion self-care (e.g., being compliant with hospital discharge instructions) than their male counterparts. However, five studies [15,24,25,26,27] concluded that male older adults were more likely to adopt healthy habits.

*Age*: Eight studies [14,19,20,22,24,26,28,29] found that people with advancing age were more likely to keep up with healthy habits than their counterparts. Eight studies [16,17,18,23,27,30,31,32] had the opposite conclusion: that advancing age decreased the likelihood of performing health promotion and disease prevention behaviors.

*Marital status*: Seven articles [14,15,16,17,27,31,33] found that married older adults were more likely to engage in healthy lifestyles and disease prevention behaviors than their counterparts who were single, divorced, widowed, or separated. In contrast, the study conducted by Ang et al. found that married older adults were less likely to adopt disease prevention behaviors than their non-married counterparts [19].

*Education level*: Eleven studies [14,15,16,20,22,26,32,33,34,35,36] found that older adults with more than a high school education were more likely to engage in healthy lifestyles than their counterparts with a high school education or less. In contrast, Awad et al. found that older adults with lower education levels were more likely to engage in health promotion and disease prevention self-care behaviors [24].

These reviewed articles showed varying associations and patterns between older adults’ socio-demographic determinants and their health promotion and disease prevention-related behaviors. These review findings inform the necessity for further research on the subject, which was the present study’s aim. As summarized in the Health Affairs Blog written by Green and Zook [37], the Health Care Transformation Task Force in the U.S. claimed that social determinants of health could impact any individual. Some of these determinants could present health benefits to specific populations and cause harm to other people. These determinants are not always positive or negative [37]. In short, as an exploratory survey study, the authors did not hypothesize the relational directions between the five socio-demographic determinants of health behaviors and nine self-care behaviors related to promoting health or preventing disease.

## 2. Methods

### 2.1. Study Design and Participants

This study is a secondary data analysis from an original cross-sectional 2015–2016 survey project of community-dwelling adults living in the southern United States (as the parent study). The parent study received ethics approval from the Tennessee Technological University Institutional Review Board (IRB). The original survey study included a sample of 250 adults with a response rate of 82%. The targeted and achieved sample size was determined based on the resources available [7,9]. Convenience sampling was used. The inclusion criteria were: (1) community-dwelling adults aged 18 years and older, and (2) being able to speak and read English. There were no exclusion criteria. Participants may be healthy and may not have a medical condition. Participation was voluntary. Since this parent study did not collect personal information, no written consent was required per IRB approval. An information sheet about the study was provided to each potential participant in-person at the study sites by research assistants. Completing the survey indicated each participant’s consent to participate in the study. After completing the survey, each participant received a $5 grocery gift card. We selected the data collection locations based on the ease of access, including eight senior centers and the student health service at a local university, Tennessee Technological University [7,9]. To address potential sources of sample selection bias, the authors solicited participation across multiple senior centers and the student health service at the university. We included adults living in urban and rural areas [7,9].

The 2015–2016 survey project includes many variables (the survey tool may be obtained from the corresponding author via e-mail). The data collected in this parent study has been used in eight peer-reviewed publications. No research questions reported in these publications overlap with the ones addressed in the present study (i.e., dependent variables and associations being tested) [7,9].

For the present study, we retrieved older adults (aged 65 years and older, recruited from eight senior centers) from the original sample size for secondary analysis (*n* = 123). Note that the sample used for this present study was part of the parent study’s convenient sample. We only included the data collected in the senior centers in this current study (a convenient subsample selection to focus on older adults).

Based on a mathematical computation posited by Peduzzi et al. [38] for sample size calculations involving binary logistic regression analyses, the sample size (*n* = 123) was sufficient for univariate logistic regression analyses. Peduzzi et al. [38] used the following guideline for sample size calculations (a minimum number of cases): Let p be the smallest of the proportions of negative or positive cases in the population and k the number of covariates (the number of independent variables), then the minimum number of cases to include is: *n* = 10 k/p. Thus, the sample size was insufficient for conducting multivariate logistic regression analyses, which would require a minimum sample of *n* = 400. As a supplemental analysis, we performed multiple logistic regression analyses to explore possible relationships when all demographic-social determinants were entered into the regression models simultaneously.

### 2.2. Data Collection Instruments

We used two self-administered survey instruments: (1) the Patient Action Inventory for Self-Care questionnaire and (2) a demographic questionnaire [9]. The Patient Action Inventory for Self-Care questionnaire is an 11-category behavior assessment tool, which has been validated for construct validity and reliability in the adult population by Tzeng and Pierson [9]. This tool uses dichotomous responses (yes or no) to measure the perception of the importance of 57 delineated self-care behaviors, their desire, and their ability to perform these behaviors [9]. The self-care areas (promoting health and getting preventive health care) focused in this present study include two categories of self-care behaviors; these two categories have nine self-care behaviors.

For the present study, the authors collected responses of nine self-care behaviors from the Patient Action Inventory for Self-Care questionnaire. These nine self-care items are: (#1) creating habits that will improve health and prevent disease; (#2) finding and using services that support your health behaviors; (#3) keeping your new health behaviors going; (#4) following the agreed treatment plan to manage your symptoms; (#5) discussing the use of health screening tests with your provider; (#6) seeking early detection of diseases, like cancer; (#7) following up on health screening results; (#8) getting needed vaccines; and (#9) joining in local health screening or wellness events. Self-care items #1 through #4 are grouped under the theme heading “promoting health,” and items #5 through #9 are grouped under the theme heading “getting preventive health care” in the inventory. Respondents answered yes or no (1 = yes, 0 = no) based on their self-perception of which self-care items they consider being (1) important, (2) desirable, and (3) within their ability to perform.

In the demographic questionnaire, five demographic characteristics were included in this study: residence (1 = urban, 0 = rural), gender (1 = male, 0 = female), age (1 = 65 years to <75 years, 2 = 75 years to <85 years, 3 = 85 years and older), marital status (1 = married; 2 = single, divorced, or widowed; 3 = separated), and education level (1 = less than high school diploma; 2 = high school diploma; 3 = associate’s degree, bachelor’s degree, or higher).

### 2.3. Data Analysis

Data were analyzed using the IBM Statistical Package for Social Sciences (SPSS) 26.0 statistical software (IBM Corp., Armonk, NY, USA). All data (from completed or partially completed returned surveys) were included in the analysis. Categorical variables were described using frequencies and percentages. Related to the missing values, we did not perform data manipulation for the model simulation. We excluded the cases with missing values for analysis.

We conducted univariate logistic regression analyses for each of the nine self-care questions by (1) the importance, (2) desirability, and (3) ability to perform, with each of the five demographic characteristics to assess each demographic characteristic’s contribution. As supplementary analyses, we conducted multivariate logistic regression analyses (method = enter) to determine the contributions of all five demographic attributes to the perception of importance, desire to perform, and ability to perform each of the nine self-care behaviors. We set the significance level (alpha) for all statistical tests at 0.05, two-sided.

## 3. Results

The descriptive analyses of variables are reported in Table 1. Of the 123 older adult respondents, 90 (73.3%) were female, and 76 (61.8%) lived in rural counties. Sixty (48.8%) were between the ages of 65 and ≤75 years, 49 (39.9%) were single, and 82 (66.7%) had a high school diploma. Findings of univariate and supplementary analyses are summarized below.

### 3.1. Univariate Logistic Regression Analyses

We found four models with at least one significant regression coefficient at *p*-value < 0.05. Supplemental Table S1 summarizes four significant univariate models. As shown in Supplemental Table S1, three of them were related to self-care item #5, discussing the use of health screening tests with a healthcare provider. Older adults ≥85 years were less likely to consider it important to discuss the use of health screening tests with their providers than their counterparts between the ages of 65 and <75 years (OR = 0.08; *p*-value = 0.035; 95% CI = 0.008 to 0.833). Older adults with an associate degree or higher were more likely (more than 4 folds with OR of 4.58) to express the desire to discuss the use of health screening tests with their providers compared with their counterparts with less than a high school education (OR = 4.58; *p*-value = 0.037; 95% CI = 1.093 to 19.217). Older adults with a high school diploma were more likely (more than 14 folds) to have the ability to discuss the use of health screening tests with their providers compared with their counterparts with less than a high school education (OR = 14.222; *p*-value = 0.037; 95% CI = 1.168 to 173.229). In addition, older separated adults were less likely to have the ability to join in local health screening or wellness events (self-care item #9) compared with their counterparts who are married (OR = 0.147; *p*-value = 0.043; 95% CI = 0.023 to 0.940).

### 3.2. Supplementary Analyses

Five multivariate logistic regression models had at least one statistically significant regression coefficient (*p*-value < 0.05) after including all five demographic variables using the enter method on SPSS (IBM Corp., Armonk, NY, USA). Supplemental Table S2 summarizes significant multivariate logistic regression models.

As shown in Model #1, older adults with a high school diploma were more likely (more than 38 folds) to report “yes” to having the desire to perform self-care action #1 (creating habits that will improve health and prevent disease) compared with their counterparts with less than a high school education (OR = 38.57; *p*-value = 0.028; 95% CI = 1.489 to 999.356). As indicated in Model #2, older adults with a high school diploma were more likely (more than 108 folds) to report “yes” to perform self-care action #5 (discussing use of health screening tests with their provider) compared with less than a high school education (OR = 108.29; *p*-value = 0.019; 95% CI = 2.156 to 5440.57).

Models #3 through #5 addressed self-care behavior #9 (joining in local health screening or wellness events). As stated in Model #3, older adults with a high school diploma were more likely (more than 4 folds) to report “yes” to perceiving it as important to perform self-care behavior #9 compared with their counterparts with less than a high school education (OR = 4.56; *p*-value = 0.048; 95% CI = 1.011 to 20.558). In Model #4, older adults with a high school diploma were more likely (more than 5 folds) to report “yes” to having the desire to perform self-care action #9 compared with their counterparts with less than a high school education (OR = 5.48; *p*-value = 0.038; 95% CI = 1.101 to 27.22). As shown in Model #5, older adults who are separated were less likely to report “yes” to perform self-care action #9 compared with their counterparts who are married (OR = 0.027; *p*-value = 0.037; 95% CI = 0.001 to 0.805).

Table 2 provides an overview of significant socio-demographic determinants with self-care items to promote health and prevent disease based on the results from the univariate and multivariate logistic regression analyses.

## 4. Discussion

This exploratory study investigated the relationships between five socio-demographic determinants and the perception of importance, desire to perform, and ability to perform nine self-care behaviors relating to promoting health and getting preventive services among community-dwelling older adults living in southern United States. We found three socio-demographic determinants (advancing age, marital separation, and holding less than a high school education) were linked to older adults’ unfavorable perceptions of three health promotion and disease prevention-related self-care behaviors. These three behaviors were: (1) creating habits that will improve health and prevent disease, (2) discussing the use of health screening tests with their healthcare providers, and (3) joining in local health screening or wellness events.

Separated older adults and those with less than a high school education were less likely to perform the disease prevention-related self-care behaviors than their counterparts (i.e., discussing the use of health screening tests with their healthcare providers, and joining in local health screening or wellness events). Older adults with advancing age and less than a high school education were less inclined to value the importance of and express their desire to perform all three self-care behaviors of (1) creating habits that will improve health and prevent disease, (2) discussing the use of health screening tests with their healthcare providers, and (3) joining in local health screening or wellness events. These findings imply that the healthcare and social service sectors should not take a one-size-fits-all approach when addressing older adults’ self-care behaviors. Our study found that older adults with more education were more likely to engage in healthy lifestyles than their counterparts with less education. This finding was consistent with the conclusion of several previous studies [14,15,16,20,22,26,32,33,34,35,36].

As for the two “getting preventive healthcare” self-care behaviors—discussing the use of health screening tests with healthcare providers (item #5) and joining in local health screening or wellness events (item #9)—our findings were consistent with several previous studies [16,17,18,23,27,30,31,32]. These studies found that advancing age decreased the likelihood of performing health promotion and disease prevention behaviors. A few published studies [14,15,16,17,27,31,33] also found that married older adults were more likely to engage in healthy lifestyles and disease prevention behaviors than their non-married counterparts. Furthermore, several previous studies [14,15,16,20,22,26,32,33,34,35,36] concluded that older adults with more education were more likely to engage in healthy lifestyles than their counterparts with less education. Specific to the self-care behavior of joining in local health screening or wellness events (item #9), social participation in wellness events has been reported in previous studies to provide health benefits to older adults, including but not limited to improved physical health (e.g., physical strength building to decrease the development of disabilities), mental health (e.g., reduced depressive symptomatology and improved memory function), and social health, as well as increased life expectancy [39,40,41,42,43].

### 4.1. Practical Implications

Clinicians in all care settings should consider adopting a patient-centered care delivery model or approach and routinely incorporate demographic and social risk data into older adults’ health care decisions [4]. We suggest researchers include social determinants of health when examining patient outcomes. Our findings also suggest that educators emphasize the importance of social determinants of health and the necessity to engage patients in their own health decisions to improve their health optimally. It is also essential for policymakers and funding agencies to strategically fund research and evaluation on the effectiveness of health care and social service sectors in promoting health among community-dwelling older adults and getting them to adopt preventive health care [4].

### 4.2. Study Limitations and Future Research Directions

The study findings may not be generalizable to other settings with varying socio-ethnic and cultural inclinations. This survey study did not assess participants’ health status and did not validate participants’ self-reported health status (e.g., via chart review) as a limitation. This study’s sample size is limited (*n* = 123 or less due to missing responses, which vary from variable to variable), which is sufficient for conducting binary logistic regression analyses based on sample size calculations by Peduzzi et al. [38]. The work of Peduzzi et al. [38] suggested the following guideline for a minimum number of cases: Let p be the smallest of the proportions of negative or positive cases in the population and k the number of covariates (the number of independent variables), then the minimum number of cases to include is: *n* = 10 k/p.

Unfortunately, this study does not have a sufficient sample size to run multiple logistic regression. There were eight regression coefficients in the multiple logistic regression model. It is supposed that the proportion of favorable outcome is 0.20 (20%), and the minimum number of cases required is *n* = 10 × 8/0.20 = 400. This study did not have a sufficient sample size for multiple logistic regression with eight regression coefficients (demographic characteristics). Due to low sample size and ultimately low power, this study could commit a Type 2 error (a type II error occurs when we declare no differences or associations when, in fact, there was.) [44]. As a result, result interpretation for binary and multiple logistic regression analyses should be cautious.

The exact sample size varied due to missing responses, which vary from a binary dependent variable to another variable. Due to the missing answers, this survey study could also commit a Type I error when we reject the null hypothesis and erroneously state that the study found significant differences when there was no difference [44]. We treated missing responses as system missing as the respondents chose whether they wanted to answer any self-care behavior questions. The missing answers (recorded as “no answer” in Table 1) were not replaced by yes or no. Due to the limited sample size and missing values, this study focuses on binary logistic regression findings. We conducted multivariate logistic regression as a supplemental analysis for references. As another study limitation, this secondary data analysis included limited socio-demographic characteristics. This limitation is due to the ones available in the parent survey study.

Future research may conduct interviews or focus groups with older adults to learn their insights on how the health care and social service sectors could address their needs to perform health promotion and disease prevention self-care actions. Triangulating older adults’ perspectives with (a) the community services available to them in their local regions and (b) the views of health care and social service policymakers, decision-makers, and providers could identify the gaps. These data could also help address the challenges encountered by older adults who struggle to navigate healthcare systems. This approach may also facilitate a co-designing process to identify practical solutions.

## 5. Conclusions

This exploratory study examined the perceptions of community-dwelling older adults living in southern United States toward nine health promotion and disease prevention-related self-care behaviors needed to navigate the healthcare system. Three socio-demographic determinants (i.e., advancing age, being separated compared with being married, and holding less than a high school education) were significantly associated with less favorable perceptions of the self-care behaviors of (1) creating habits that will improve health and prevent disease, (2) discussing the use of health screening tests with their healthcare providers, and (3) joining in local health screening or wellness events. Older adults’ gender and residence did not significantly associate with health promotion and disease prevention-related self-care behaviors. The findings suggested that comprehensive health care should include understanding each older adult’s socio-demographic context and related needs. It is essential to engage older adults, community partners, clinicians, and unlicensed workers in designing and implementing integrated health care and social service systems that incorporate older adults’ preferences and their residing communities.

## Figures and Tables

**Table 1:** Summary of descriptive analyses of study variables (*n* = 123).

**Demographic Variable**	**Categories (Coding for Analyses)**	**Frequency (%)**
Residential Site	Urban counties (1) Rural counties (0)	47 (38.2) 76 (61.8)
Gender	Female (0)	90 (73.3)
	Male (1) No answer (missing)	23 (18.7) 10 (8.1)
Age in years	65 to <75 years (1)	60 (48.8)
	75 to <85 years (2)	44 (35.8)
	85 years and older (3)	19 (15.4)
Marital status	Married (1)	48 (39.0)
	Single (2)	49 (39.9)
	Separated (3) No answer (missing)	12 (9.8) 14 (11.4)
Education	Less than a high school diploma (1)	18 (14.6)
	High school diploma (2)	82 (66.7)
	Associate degree, bachelor’s degree, and above (3)	23 (18.7)
Ethnic group	White, non-Hispanic	111 (90.2)
	White, Hispanic	6 (4.9)
	Black or African American	1 (0.8)
	American Indian or Alaska Native	5 (4.1)
	Asian	0 (0)
	Native Hawaiian or other Pacific Islander	0 (0)
	Other race	0 (0)
**Patients’ perceived importance levels**	**Categories (coding for analyses)**	**Frequency (%)**
(#1) Create habits that will improve health and prevent disease	No (0) Yes (1) No answer (missing)	4 (3.3) 113 (91.9) 6 (4.9)
(#2) Find and use services that support your health behaviors	No (0) Yes (1) No answer (missing)	1 (0.8) 108 (87.8) 14 (11.4)
(#3) Keep your new health behaviors going	No (0) Yes (1) No answer (missing)	0 (0.0) 113 (91.9) 10 (8.1)
(#4) Follow the agreed treatment plan to manage your symptoms	No (0) Yes (1) No answer (missing)	1 (0.8) 115 (93.5) 7 (5.7)
(#5) Discuss the use of health screening tests with your provider	No (0) Yes (1) No answer (missing)	7 (5.7) 111 (91.9) 5 (4.1)
(#6) Seek early detection of diseases, like cancer	No (0) Yes (1) No answer (missing)	3 (2.4) 113 (91.9) 7 (5.7)
(#7) Follow up on health screening results	No (0) Yes (1) No answer (missing)	3 (2.4) 111 (90.2) 9 (7.3)
(#8) Get needed vaccines	No (0) Yes (1) No answer (missing)	2 (1.6) 114 (92.7) 7 (5.7)
(#9) Join in local health screening or wellness events	No (0) Yes (1) No answer (missing)	24 (19.5) 90 (73.2) 9 (7.3)
**Patients’ perceived desire levels**	**Categories (coding for analyses)**	**Frequency (%)**
(#1) Create habits that will improve health and prevent disease	No (0) Yes (1) No answer (missing)	9 (7.3) 84 (8.3) 30 (24.4)
(#2) Find and use services that support your health behaviors	No (0) Yes (1) No answer (missing)	7 (5.7) 79 (64.2) 37 (30.1)
(#3) Keep your new health behaviors going	No (0) Yes (1) No answer (missing)	4 (3.3) 86 (69.9) 33 (26.8)
(#4) Follow the agreed treatment plan to manage your symptoms	No (0) Yes (1) No answer (missing)	5 (4.1) 84 (68.3) 34 (27.6)
(#5) Discuss the use of health screening tests with your provider	No (0) Yes (1) No answer (missing)	10 (8.1) 81 (65.9) 32 (26.0)
(#6) Seek early detection of diseases, like cancer	No (0) Yes (1) No answer (missing)	8 (6.5) 85 (69.1) 33 (26.8)
(#7) Follow up on health screening results	No (0) Yes (1) No answer (missing)	5 (4.1) 85 (69.1) 28 (22.8)
(#8) Get needed vaccines	No (0) Yes (1) No answer (missing)	9 (7.3) 86 (69.9) 28 (22.8)
(#9) Join in local health screening or wellness events	No (0) Yes (1) No answer (missing)	24 (19.5) 68 (55.3) 31 (25.2)
**Patients’ perceived ability levels**	**Categories (coding for analyses)**	**Frequency (%)**
(#1) Create habits that will improve health and prevent disease	No (0) Yes (1) No answer (missing)	4 (3.3) 95 (77.2) 24 (19.5)
(#2) Find and use services that support your health behaviors	No (0) Yes (1) No answer (missing)	4 (3.3) 87 (70.7) 32 (26.0)
(#3) Keep your new health behaviors going	No (0) Yes (1) No answer (missing)	2 (1.6) 96 (78.0) 25 (20.3)
(#4) Follow the agreed treatment plan to manage your symptoms	No (0) Yes (1) No answer (missing)	2 (1.6) 96 (78.0) 25 (20.3)
(#5) Discuss the use of health screening tests with your provider	No (0) Yes (1) No answer (missing)	6 (4.9) 91 (74.0) 26 (21.1)
(#6) Seek early detection of diseases, like cancer	No (0) Yes (1) No answer (missing)	3 (2.4) 90 (73.2) 30 (24.4)
(#7) Follow up on health screening results	No (0) Yes (1) No answer (missing)	4 (3.3) 90 (73.2) 29 (23.6)
(#8) Get needed vaccines	No (0) Yes (1) No answer (missing)	2 (1.6) 97 (78.9) 24 (19.5)
(#9) Join in local health screening or wellness events	No (0) Yes (1) No answer (missing)	11 (8.9) 86 (69.9) 26 (21.1)

**Table 2:** Summary of the logistic regression analyses of perceived importance, desire to perform, and ability to perform nine patient engagement self-care actions for “promoting health” and “getting preventive health care.” We indicated significant associations in the corresponding boxes with the demographic characteristics. The level of significance, alpha, was set at 0.05 for two-sided statistical tests.

Univariate Logistic Regression (Including Only One Demographic Characteristic in the Model)			
Getting Preventive Healthcare/Levels ^a^	Importance Level	Desire Level	Ability Level
(#5) Discuss the use of health screening tests with your provider	Older adults aged 85 years and above were less likely to respond positively than older adults within the age group of 65 to <75.	Older adults who had an associate degree or bachelor’s degree were more likely to have a positive response than older adults with less than a high school education level.	Older adults who had an associate degree or bachelor’s degree were more likely to have a positive response than older adults with less than a high school education level.
(#9) Join in local health screening or wellness events	--	--	Separated older adults were less likely to positively respond to this self-care behavior than married older adults did.
**Supplementary Analysis:** **Multiple Logistic Regression (Including all Five Demographic Characteristics in the Same Model)**			
Promoting health/Levels ^a^	Importance level	Desire level	Ability level
(#1) Create habits that will improve health and prevent disease	--	Older adults with a high school diploma were more likely to have a positive response to this self-care behavior compared with older adults with less than a high school level of education	--
Getting preventive healthcare/Levels ^a^	Importance level	Desire level	Ability level
(#5) Discuss the use of health screening tests with your provider	--	--	Older adults with a high school diploma were more likely to positively respond to this self-care behavior than older adults with less than a high school education level.
(#9) Join in local health screening or wellness events	Older adults with a high school diploma were more likely to positively respond to this self-care behavior than older adults with less than a high school education level.	Older adults with a high school diploma were more likely to positively respond to this self-care behavior than older adults with less than a high school education level.	Separated older adults were less likely to positively respond to this self-care behavior than married older adults did.

^a^: A positive response = Yes to being important, desirable to perform, or able to perform.

## Data Availability

The data used for this present study may be obtained from the corresponding author via e-mail.

## Supplementary Materials

The following are available online at https://www.mdpi.com/2039-4403/11/1/12/s1, Table S1: Summary of the univariate logistic regression analyses. Table S2: Summary of multiple logistic regression models with at least one statistically significant regression coefficient value. Five models showed the statistical significance summarized in this table.
