# Supplementary material for: Older Adults’ Socio-Demographic Determinants of Health Related to Promoting Health and Getting Preventive Health Care in Southern United States: A Secondary Analysis of a Survey Project Dataset
Source: Nurs Rep. 2021 Feb 15;11(1):120–32. doi: 10.3390/nursrep11010012 (PMC8009605; doi:10.3390/nursrep11010012)
Supplement: Supplementary file 1 [file nursrep-11-00012-s001.pdf]

Table S1. Summary of the univariate logistic regression analyses.

Model 1: Univariate logistic regression predicting the likelihood of reporting yes (1) to the perceived importance of self-care action to discuss the use of health screening tests with your provider (action item #5)

	B	S.E.	Wald	df	p	Odds Ratio	95% C.I. for Odds Ratio/Lower	95% C.I. for Odds Ratio/Upper
Ref= (65 to <75 years)			4.611	2	0.100			
Age (75 to < 85years)	−1.495	1.173	1.625	1	0.202	0.224	0.022	2.234
Age (85 years and above)	−2.520	1.192	4.466	1	0.035 *	0.080	0.008	0.833

*: $p < 0.05$.

Model 2: Univariate logistic regression predicting the likelihood of reporting yes (1) to desire to perform self-care action to discuss the use of health screening tests with your provider (action item #5)

	B	S.E.	Wald	df	p	Odds Ratio	95% C.I. for Odds Ratio/Lower	95% C.I. for Odds Ratio/Upper
Ref= (Less than high school)			4.572	2	0.102			
Education (High school diploma)	1.299	1.165	1.243	1	0.265	3.667	0.374	35.979
Education (Associate degree, bachelor's degree, and above)	1.522	0.731	4.334	1	0.037 *	4.583	1.093	19.217

*: $p < 0.05$.

Model 3: Univariate logistic regression predicting the likelihood of reporting yes (1) to the ability to perform self-care action to discuss the use of health screening tests with your provider (action item #5)

	B	S.E.	Wald	df	p	Odds Ratio	95% C.I. for Odds Ratio/Lower	95% C.I. for Odds Ratio/Upper
Ref= (Less than high school)			4.986	2	0.083			
Education (High school diploma)	2.655	1.275	4.333	1	0.037 *	14.222	1.168	173.229
Education (Associate degree, bachelor's degree, and above)	0.288	1.000	0.083	1	0.774	1.333	0.188	9.465

*: $p < 0.05$.

Model 4: Univariate logistic regression predicting the likelihood of reporting yes (1) to the ability to perform self-care action to join in local health screening or wellness events (action item #9)

	B	S.E.	Wald	df	p	Odds Ratio	95% C.I. for Odds Ratio/Lower	95% C.I. for Odds Ratio/Upper
Ref= (Married)			4.831	2	0.089			
Marital status (Single)	−0.176	0.799	0.049	1	0.825	0.838	0.175	4.016
Marital status (Separated)	−1.917	0.947	4.101	1	0.043*	0.147	0.023	0.940

*: $p < 0.05$.

Table S2. Summary of multiple logistic regression models with at least one statistically significant regression coefficient value. Five models showed the statistical significance summarized below.

Model #1: Predicting the likelihood of reporting yes (1) to perceived desire to perform self-care action #1 (Create habits that will improve health and prevent disease)

	B	S.E.	Wald	df	Sig.	Odds Ratio	95% C.I. for Odds Ratio/Lower	95% C.I. for Odds Ratio/Upper
Residence (1 = urban) Ref = Rural	−1.220	1.059	1.328	1	0.249	0.295	0.037	2.351
Gender (1 = male) Ref = Female	19.951	9669.476	0.000	1	0.998	461889617.2	0.000	.
Ref= (65 to <75 years)			1.283	2	0.527			
Age (75 to < 85 years)	0.551	1.248	0.195	1	0.659	1.734	0.150	20.033
Age (85 years and above)	1.947	1.724	1.274	1	0.259	7.006	0.239	205.741
Ref= (Married)			3.332	2	0.189			
Marital status (Single)	−2.891	1.618	3.191	1	0.074	0.056	0.002	1.324
Marital status (Separated)	−2.555	1.737	2.165	1	0.141	0.078	0.003	2.337
Ref= (Less than high school)			5.563	2	0.062			
Education (High school diploma)	3.653	1.661	4.838	1	0.028*	38.572	1.489	999.356
Education (Associate degree, bachelor's degree, and above)	1.458	1.684	0.749	1	0.387	4.297	0.158	116.655

*: $p < 0.05$.

Model #2: Predicting the likelihood of reporting yes (1) to being able to perform self-care action #5 (Discuss use of health screening tests with your provider)

	B	S.E.	Wald	df	Sig.	Odds Ratio	95% C.I. for Odds Ratio/Lower	95% C.I. for Odds Ratio/Upper
Residence (1 = urban) Ref = Rural	−0.888	1.415	0.394	1	0.530	00.412	0.026	6.585
Gender (1 = male) Ref = Female	−1.950	1.475	1.747	1	0.186	0.142	0.008	2.565
Ref= (65 to <75 years)			2.708	2	0.258			
Age (75 to < 85years)	−3.393	2.062	2.708	1	0.100	0.034	0.001	1.913
Age (85 years and above)	18.081	9854.817	0.000	1	0.999	71184464.6	0.000	.
Ref= (Married)			0.385	2	0.825			
Marital status (Single)	−0.939	1.515	0.384	1	0.535	0.391	0.020	7.623
Marital status (Separated)	−0.387	2.042	0.036	1	0.850	0.679	0.012	37.131
Ref= (Less than high school)			5.681	2	0.058			
Education (High school diploma)	4.685	1.998	5.496	1	0.019*	108.296	2.156	5440.577
Education (Associate degree, bachelor's degree, and above)	1.089	1.983	0.302	1	0.583	2.972	0.061	144.840

*: $p < 0.05$.

Model #3: Predicting the likelihood of reporting yes (1) to the perceived importance of self-care action #9 (Join in local health screening or wellness events)

	B	S.E.	Wald	df	Sig.	Odds Ratio

							95% C.I. for Odds Ratio/Lower	95% C.I. for Odds Ratio/Upper
Residence (1 = urban) Ref = Rural	−0.719	0.581	1.533	1	0.216	0.487	0.156	1.521
Gender (1 = male) Ref = Female	1.189	0.865	1.887	1	0.170	3.283	0.602	17.897
Ref= (65 to <75 years)			0.428	2	0.807			
Age (75 to < 85years)	−0.095	0.668	0.020	1	0.887	0.910	0.246	3.369
Age (85 years and above)	−0.501	0.778	0.414	1	0.520	0.606	0.132	2.783
Ref= (Married)			0.849	2	0.654			
Marital status (Single)	−.528	0.609	0.751	1	0.386	0.590	0.179	1.946
Marital status (Separated)	−0.555	0.899	0.382	1	0.537	0.574	0.099	3.342
Ref= (Less than high school)			4.573	2	0.102			
Education (High school diploma)	1.517	0.768	3.897	1	0.048*	4.559	1.011	20.558
Education (Associate degree, bachelor's degree, and above)	0.656	0.945	0.481	1	0.488	1.926	0.302	12.282

*: $p < 0.05$.

Model #4: Predicting the likelihood of reporting yes (1) to perceived desire to perform self-care action #9 (Join in local health screening or wellness events)

	B	S.E.	Wald	df	Sig.	Odds Ratio	95% C.I. for Odds Ratio/Lower	95% C.I. for Odds Ratio/Upper
Residence (1 = urban) Ref = Rural	−1.186	0.635	3.493	1	0.062	0.305	0.088	1.059
Gender (1 = male) Ref = Female	0.002	0.860	0.000	1	0.998	1.002	0.186	5.410
Ref= (65 to <75 years)			0.077	2	0.962			
Age (75 to < 85years)	−0.200	0.726	0.076	1	0.783	0.819	0.197	3.397
Age (85 years and above)	−0.075	0.853	0.008	1	0.930	0.927	0.174	4.938
Ref= (Married)			2.216	2	0.330			
Marital status (Single)	−0.874	0.639	1.870	1	0.171	0.417	0.119	1.460
Marital status (Separated)	−1.188	1.050	1.280	1	0.258	0.305	0.039	2.388
Ref= (Less than high school)			4.336	2	0.114			
Education (High school diploma)	1.700	0.818	4.318	1	0.038*	5.475	1.101	27.218
Education (Associate degree, bachelor's degree, and above)	1.424	1.081	1.735	1	0.188	4.154	.499	34.586

*: $p < 0.05$.

Model #5: Predicting the likelihood of reporting yes (1) to being able to perform self-care action #9 (Join in local health screening or wellness events)

	B	S.E.	Wald	df	Sig.	Odds Ratio	95% C.I. for Odds Ratio/Lower	95% C.I. for Odds Ratio/Upper
Residence (1 = urban) Ref = Rural	−1.918	1.286	2.223	1	0.136	0.147	0.012	1.828

Gender (1 = male) Ref = Female	−1.254	1.497	0.702	1	0.402	0.285	0.015	5.362
Ref= (65 to <75 years)			0.487	2	0.784			
Age (75 to < 85years)	−19.857	5377.370	0.000	1	0.997	0.000	0.000	.
Age (85 years and above)	−20.632	5377.370	0.000	1	0.997	0.000	0.000	.
Ref= (Married)			4.699	2	0.095			
Marital status (Single)	−0.014	1.124	0.000	1	0.990	0.986	0.109	8.923
Marital status (Separated)	−3.608	1.730	4.350	1	0.037 *	0.027	0.001	0.805
Ref= (Less than high school)			3.247	2	0.197			
Education (High school diploma)	2.429	1.391	3.048	1	0.081	11.350	0.742	173.518
Education (Associate degree, bachelor’s degree, and above)	2.831	1.970	2.067	1	0.151	16.969	0.357	805.727

*: $p < 0.05$.
